# Change in pain, disability and influence of fear-avoidance in a work-focused intervention on neck and back pain: a randomized controlled trial

**DOI:** 10.1186/s12891-015-0553-y

**Published:** 2015-04-21

**Authors:** Gunn Hege Marchand, Kjersti Myhre, Gunnar Leivseth, Leiv Sandvik, Bjørn Lau, Erik Bautz-Holter, Cecilie Røe

**Affiliations:** Department of Neuroscience, Faculty of Medicine, Norwegian University of Science and Technology, Trondheim, Norway; Department of Physical Medicine and Rehabilitation, St. Olav’s Hospital, Trondheim University Hospital, Trondheim, Norway; Department of Physical Medicine and Rehabilitation, Oslo University Hospital, Ulleval, Oslo Norway; Institute of Clinical Medicine, Neuromuscular Disorders Research Group, UiT The Arctic University of Norway, Tromsø, Norway; Department of Biostatistics and Epidemiology, Oslo University Hospital, Ulleval, Oslo Norway; Lovisenberg Diakonale Hospital, Oslo, Norway; National Institute of Occupational Health, Oslo, Norway; Faculty of Medicine, University of Oslo, Oslo, Norway

**Keywords:** Low back pain, Neck pain, Fear avoidance beliefs, Work disability, Disability, Return to work, Multidisciplinary intervention, Brief intervention

## Abstract

**Background:**

Neck and back pain are among the most common causes of prolonged disability, and development of interventions with effect on pain, disability and return to work is important. Reduction of fear avoidance might be one mechanism behind improvement after interventions. The aim of the present study was to evaluate changes in pain and disability at the 12-month follow-up of patients with neck and back pain treated with a work-focused intervention compared to patients treated with standard interventions, and the influence of improvement fear avoidance beliefs during the interventions on pain, disability and return to work at 12-month follow-up.

**Methods:**

413 employed patients with back or neck pain referred to secondary care, and sick-listed between 4 weeks and 12 months, were randomized to a work-focused rehabilitation or control interventions. Follow-up was conducted 4 and 12 months after inclusion. The groups were compared (independent sample t-test) regarding differences in disability scores (Oswestry disability index/neck disability index) and pain (numeric rating scale) from baseline to 12-month follow-up. Changes in fear avoidance beliefs (FABQ) from baseline to 4 month follow-up were calculated, and the association between this change and return to work, pain and disability at 12 months were tested in stepwise multiple logistic regression models.

**Results:**

Pain and, disability scores decreased to in both the work-focused and control intervention to 12-month follow-up, and there were no significant differences between the groups. FABQ decreased similarly in both groups to 4 month follow-up. The logistic regression model revealed an association between a reduced FABQ work score at 4 months and return to work within one year (adjusted OR 3.60, 95% CI 1.19 to 10.88). Reduced FABQ physical activity score at 4 months was associated with decreased disability after 12 months (adjusted OR (3.65. 95% CI 1.43 to 9.28).

**Conclusions:**

Short work-focused rehabilitation had the same effect on pain and disability as control interventions. Reduction in FABQ-W score after treatment seems to be an important predictor for return to work in both groups.

**Trial registration:**

Clinicaltrials.gov NCT00840697

## Background

Neck and back pain are among the most common causes of prolonged disability in “the global burden of disease study” [1,2], and development of effective rehabilitation strategies is of major importance. Various rehabilitation programs that aim to reduce pain and improve functional status currently exist [3-7]. Models that more specifically target the return to work (RTW) process have also been developed [8-10]. The effectiveness of some work- focused interventions has been found to be superior to control interventions regarding RTW [10-13], without differences on pain and disability. Even in subjects with chronic low back pain who are assumed to be at a higher risk for not returning to work, a work focused intervention improved RTW rates more than the control intervention [12]. In contrast, Jensen et al. found no differences between work-focused and brief interventions on any outcome (pain, disability, RTW) [14]. In a previous published paper from our study, analysing RTW as primary outcome, we also found no difference in RTW between a work-focused intervention and a control intervention [15]. The control interventions used for comparison to work-focused programs ranged in intensity from usual care [12,16] and brief interventions [14], to multidisciplinary interventions sequentially following the work-focused interventions [10,11].

A general methodological challenge in these interventions studies is that self-reported pain and disability may be flawed by a lack of response at follow-up, whereas RTW rates can be reliably determined through register-based data collection. Missing data compromise the ability to perform intention-to-treat analyses in randomised trials [17], and because the risk factor profiles of non-responders may differ from those of responders, the effect of risk factors is not easily predicted [18]. Another shortcoming in previous work-focused interventions is that only one studied patients in secondary care, despite the major contribution of this patient group to sick-leave and disability costs [19].

The mechanism behind improvement in the different interventions is complex [20]. Believing in the vulnerability of the spine and the need to avoid activities (Fear avoidance beliefs) seems to be one of the strongest predictors of prolonged pain, delayed recovery, as well as work absence [21-26]. Wertli et al. reviewed the predictive, mediating and moderating role of fear avoidance beliefs (FAB) on treatment outcome. The predictive value, evaluated by the Fear Avoidance Beliefs Questionnaire (FABQ) or the Tampa scale [27,28], varied across studies. Six out of ten studies found moderating effects of FABQ. Only four studies evaluated the mediating effects of FABQ. In all of these, reduction in FAB was associated with improved outcome [29-31]. The effects on pain and RTW were mediated by both the physical subscale (FABQ-P) and the work subscale (FABQ-W) from FABQ. Disability was influenced by FABQ-P but not by FABQ-W in acute and subacute low back pain [32], but no influence was found for FABQ-P or FABQ-W on disability in chronic low back pain patients [30]. Only one of the studies was conducted in secondary care [29]. However, the type of interventions addressing fear avoidance varied greatly across studies [33], and none of these were work-focused. Hence, we lack evidence for the influence of work- focused interventions on FABs and the related consequences for outcome. It is also unclear if the physical- and work-related components might influence pain, disability and RTW differently.

Thus, the first aim of the present study was to report secondary outcomes of a randomized controlled trial where we compared the effect of a work-focused intervention with control interventions in patients referred to secondary care for neck and back pain. The secondary outcomes were self-reported pain and disability. Second, we wanted to assess whether changes in fear avoidance beliefs were different in work-focused and control interventions and to what extent improvement in FABQ-P and FABQ-W influenced pain, disability and return to work at 12-month follow-up.

## Methods

This study was part of a large randomised controlled multicentre trial of sick-listed patients referred to the neck and back outpatient clinics at St. Olav’s Hospital and Oslo University Hospital, Ullevål between August 2009 and August 2011. The primary outcome, RTW, was reported in Spine [15]. The patients were randomised in blocks to a work-focused or control intervention using a website hosted by the medical faculty. The allocation was concealed in the data files for the researchers analysing the outcome until all analyses were run. An independent statistician generated the block size stratified by centre. The block size was concealed for all involved in the study. The first block was 20 and subsequent blocks 10.

All patients included in the study had signed an informed consent. The study was conducted in accordance with the Helsinki Declaration and the Norwegian guidelines authorised by the Data Protection for Research at Oslo University Hospital (1207–091208). The study was evaluated by the Regional Committees for Medical and Health Research Ethics in South-East Norway (S09024b 2009/1000) and registered at clinicaltrials.gov (NCT00840697).

### Participants

All patients referred for diagnostic consideration or multidisciplinary treatment of neck and/or back pain were screened for eligibility at their first consultation at the outpatient clinic. The inclusion criteria were: neck and/or back pain, age 18–60 years, employed or self-employed, and duration of sick leave between 4 weeks and 12 months. Patients in need of surgical treatment were excluded from the study. Additional exclusion criteria were: cauda equina syndrome; symptomatic spinal deformity; osteoporosis with fracture; inflammatory rheumatic disease; pregnancy; legal labour dispute; insufficient Norwegian language skills; cardiac, pulmonary, or metabolic disease with functional restrictions; and DSM-V-diagnosed mental disorders.

### Procedures and interventions

Both work-focused and control interventions took place at the neck and back clinics of the respective hospitals, but separate teams was used for the different interventions to avoid contamination. All participants received a standard clinical examination by a physician before inclusion in the study. In this consultation relevant imaging was evaluated and patients were informed about findings and were also informed that the origin of pain is often difficult to visualise via imaging. Patients were also reassured that daily activities, physical exercise, or work would not hurt or damage their necks or backs. Emphasis was placed on removing fear avoidance, restoring activity level, and enhancing self-care and coping.

At the time of this study, the neck and back clinic at St. Olav’s hospital used a comprehensive multi-disciplinary intervention based on the model described by Brox et al. [34], whereas the neck and back clinic at Oslo University Hospital used a brief model based on the model by Indahl et al. [35]. Both programmes were used as control interventions (Table 1). The brief intervention at Oslo University hospital consisted of the diagnostic clarification at the first visit and a session with a physiotherapist. The physiotherapist advised patients in activities and encouraged the patients to exercise. The physiotherapists also focused on reducing fear avoidance. One clarifying session with the medical specialist was also offered within 2 weeks.

**Table 1:** **Interventions**

	**Work**-**focused intervention**		**Control interventions**	
	**Oslo**	**Trondheim**	**Oslo**	**Trondheim**
Team	Multi-disciplinary health care professionals			
	Case worker	Case worker		
Total Duration of intervention	3 weeks	3 weeks	3 weeks	3 weeks
Sessions with physiotherapist	7	7	1-2	17
Lectures	4	5	0	8
Group discussions	0	3	0	4
No. of appointments with a medical specialist	2	2	1	2
No. of appointments with case worker	2(−3)	2	0	0

The multidisciplinary intervention at St. Olav’s hospital was administered by a team of medical specialists, physiotherapists and a social worker. The treatment had components of both cognitive behavioural therapy and exercise. The main focus was on reassurance, removing fear avoidance and physical conditioning.

The work-focused intervention was also a multidisciplinary intervention, and had duration of 5–6 days (Table 1). However, additional focus was placed on the RTW process and on reducing FAB of work. Patients received individual appointments with a caseworker during the first days of treatment. Work histories, family lives, and obstacles to RTW were discussed. The caseworkers contacted participants’ employers by phone in most cases (unless the patient refused) to inform them of the programme and to inquire about possible temporary modifications at work. The patients created a RTW schedule together with the caseworker and the multi-disciplinary team. The patients and caseworkers also discussed relevant issues for a meeting with the employer. Additionally, the caseworkers offered the patients assistance at this meeting if requested. If sick-leave compensation was an issue, the caseworkers contacted municipal social services. The medical records and RTW schedules were sent to participants and their general practitioner, who managed the patients’ sick-leave certificates.

### Data collection

The participants completed a comprehensive questionnaire before randomisation and at 4 and 12-months follow-up. Compliance to the treatment was assessed by the multidisciplinary team, and was defined as attending at least 50% of the treatment sessions offered.

#### Demographic factors

Gender and age was recorded from patient medical records. Education was classified into four categories: up to 10 years primary school; vocational high school or general academic secondary school; college or university <4 years; and college or university ≥4 years. The two last categories were collapsed for logistic regression analyses. Occupation was manually classified using the International Standard Classification of Occupations (ISCO-88) and reported using four categories: low-skilled blue collar worker, high-skilled blue collar worker, low-skilled white collar worker, and high-skilled white collar worker [36].

#### Concurrent treatment

Patients were asked if they had received treatment outside the hospital the last 4 months at the 4-month follow-up, and the last 8 months at 12-month follow-up. They answered 8 dichotomous questions (yes/no) about exercise by physiotherapist, other treatment by physiotherapist, manual therapy, psychomotor physiotherapy, treatment by chiropractor, alternative medicine, other rehabilitation programs or other therapy. If at least one question was answered “yes”, they were considered to have had concurrent treatment.

#### Hospital anxiety and depression scale (HADS)

The level of psychological distress was assessed at baseline using the validated Norwegian version of the Hospital anxiety and depression scale (HADS) [37]. HADS has one subscale for depression (HADS-D) and another for anxiety (HADS-A). Both subscales consist of 7 items scored from 0 to 3, adding up to a sum score falling within a range of 0 to 21. High scores indicate high level of symptoms. Cases with more than one missing value in a subscale were excluded. In the case of a single missing value, the missing value was replaced with the individual mean.

#### Pain

Pain was measured with an 11-point numeric rating scale (NRS) ranging from 0 (no pain) to 10 (worst possible pain) [38]. Patients were asked to rate pain at rest and during activity for back/neck pain and leg/arm pain. The highest score of the four scales was used in the analysis. Changes in scores between baseline and the 12-month follow-up were computed by subtracting the scores at 12 months from the baseline scores. In some of the analyses, these scores were dichotomised using a cut-off point of 2 for the change between baseline and 12-month follow-up [39,40].

#### Disability

Neck and back pain-related disability was measured by the Norwegian version of the Oswestry Disability Index (ODI) for back pain patients and Neck Disability Index (NDI) for neck pain patients [41,42]. Both questionnaires are composed of 10 items ranging from 0 to 5. The summed score is presented as a percentage, where 0% represents no disability and 100% represents maximum disability. In the analyses, the higher of the two scores was used if the patient had completed both questionnaires. One or two missing values were replaced with the individual mean. Scores with more than two missing values were excluded from the analysis.

Change in the ODI/NDI was calculated. In analyses with dichotomised scores, a cut-off point of 12 for the change between baseline and 12-month follow-up were applied [39,43].

#### Fear avoidance

The Fear Avoidance Belief Questionnaire (FABQ) [27] has two subscales. The physical activity subscale (FABQ-P) has four items with a possible score from 0 to 24. The work subscale (FABQ-W) has seven items with a possible score of 0 to 42. High scores indicate a high degree of fear avoidance. The minimal detectable change in the FABQ score was 9 for the physical activity subscale and 12 for the work subscale in a previous study of the Norwegian version [44]. These values were used as the cut-off in analyses with dichotomised scores.

#### Return to work

Return to work was defined as the first five-week period after randomisation that the patient did not receive sickness benefits, work assessment allowance (AAP), or disability pension from the Norwegian Labour and Welfare Administration (NAV). The five-week duration was chosen as Norwegian holidays last five weeks. Information on social compensation benefits was taken from national databases. Patients receiving partial disability pension before inclusion were considered RTW when they returned to their partly disabled status.

### Sample size

The sample size calculation was based on the primary outcome, RTW, and is reported elsewhere [15].

### Statistical methods

Comparisons of differences in baseline variables between patients who completed 12 month follow-up and patients lost to follow-up, and between the work-focused and control group were tested with t-tests for continuous variables and chi-square tests for categorical variables. Change in pain and disability from baseline to follow-up were tested using paired-sample t-tests. Differences in change scores between the work-focused and control intervention regarding pain and disability were analysed with independent samples t-tests. These analyses were carried out both with an intention to treat analyses and with available cases only (patients attending 12-month follow-up). In the intention to treat analyses multiple imputing was used to replace missing values at 12 months. Patients with missing baseline scores were not included in the analyses. The variables pain at baseline, disability at baseline, age, and return to work within one year were used to impute the missing pain scores at 12 months. The variables age, gender, education, smoking, occupation, disability at baseline, pain at baseline, and return to work within one year were used to impute disability scores at 12 months in participants with missing data at one-year follow-up. Changes in FABQ-P and FABQ-W from baseline to 4-month follow-up were calculated. Only patients with complete FABQ scores at both baseline and 4-months were included in these analyses. Subsequently, logistic regression analyses were applied to evaluate if reduction in FABQ- P and FABQ-W during the interventions influenced pain, disability and RTW within 12-month follow-up. Pain, disability and FABQ scores were dichotomised into improved and not improved in these analyses.

First, univariate logistic regression analyses, including baseline demographic variables and improvement in FABQ-P and FABQ-W from baseline to 4-month follow-up, were calculated as independent variables. Improvements in pain (≥2 points on NRS), disability (≥12 points on NDI/ODI) and RTW within 12 months were calculated as dependent variables. Subsequently, three multiple logistic regression models were built. Age, gender and intervention group were controlled for in all models and in addition we included variables with p < 0.2 from the univariate analyses. Multiple regressions controlling for baseline values of FABQ were also conducted. Correlations between independent variables were tested with Spearman’s rho, and none of the variables were correlated above 0.7. Goodness of fit was tested using the Hosmer-Lemeshow test.

A two-sided significance level of p < 0.05 was used for all analyses.

All statistical analyses were performed using SPSS Statistics, version 20 (IBM corp®, Armonk NY, USA).

## Results

**Figure 1:**
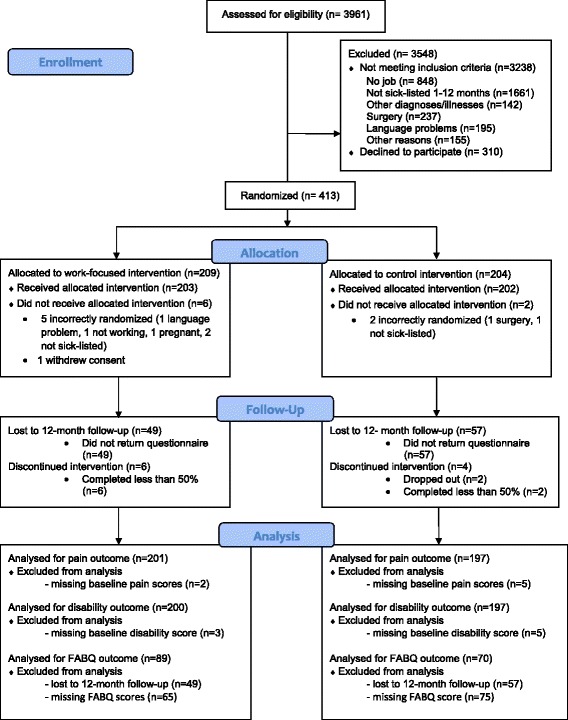
Flow chart.

A total of 723 patients were eligible for the study and 413 (57%) consented to participate. Six patients in the work-focused intervention and 2 patients in the control intervention were incorrectly randomised (Figure 1). 45 patients admitted with neck pain were included, and these were evenly distributed between the work-focused and control group. Two patients dropped out of the control intervention, but none from the work-focused intervention. Compliance was defined as accomplishing at least 50% of the treatment and 2 patients in the control intervention and 6 in the work-focused interventions were non-compliant (Figure 1).

No significant differences were found in the baseline characteristics between the participants in the work-focused intervention and the control intervention (Table 2).

**Table 2:** **Baseline characteristics**

**Variable**	**Control intervention**	**N**	**Work**-**focused intervention**	**N**
Women, n (%)	96(49%)	197	90 (45%)	201
Age (years), Mean (SD) Range (18–60)	41.08 (10.04)	197	40.09 (9.74)	201
Neck pain, n (%)	22 (11%)	197	21 (10%)	201
Norwegian mother tongue, n (%):	150 (77%)	196	160 (80%)	201
Education, n (%)		195		201
Primary school	30 (15%)		30 (15%)	
Vocational high school/general secondary school	105 (54%)		122 (61%)	
College/university <4 years	36 (19%)		31 (15%)	
College/university >4 years	24 (12%)		18 (9%)	
Occupational categories, n (%)		197		201
Low-skilled blue-collar	30 (15%)		37 (18%)	
High-skilled blue-collar	41 (21%)		46 (23%)	
Low-skilled white-collar	75 (38%)		64 (32%)	
High-skilled white-collar	51 (26%)		54 (27%)	
Smokers, n (%)	57 (29%)	195	59 (30%)	199
BMI, mean(SD) Range (16.7-45.5)	27.19 (5.03)	161	26.92 (4.68)	175
Pain, mean (SD)Range (0–10)	6.42 (2.08)	197	6.54 (2.02)	201
Disability, mean (SD)Range (8–80)	37.92 (12.88)	196	38.50 (13.78)	200
FABQ-P, mean (SD)Range (0–24)	13.74 (5.67)	192	13.85 (5.62)	196
FABQ-W, mean (SD) Range (0–42)	26.68 (10.16)	191	28.64 (9.83)	193
Depression (HAD-D), Mean (SD) Range (0–20)	5.31 (3.76)	189	5.26 (3.90)	192
Anxiety (HAD-A), Mean (SD) Range (0–18)	6.78 (3.93)	189	7.31 (4.00)	191

BMI: Body mass index, FABQ-P: Fear avoidance beliefs of physical activity, FABQ-W: Fear avoidance beliefs of work, HAD: Hospital anxiety and depression score.

Patients lost to follow-up at 12 months had higher baseline disability scores (mean difference 3.60, p = 0.018), reported higher baseline pain (mean difference 0.52, p = 0.039) and higher baseline FABQ-P scores (mean difference 1.57, p =0.015). There were also a significantly higher number of men, smokers, patients with a foreign mother tongue, and patients with low education in patients lost to follow-up. The response rate was 74 % at the 12-month follow-up. There were a similar number of patients lost to follow-up in both groups (Figure 1). Concurrent treatment (e.g. physiotherapy, manual therapy, acupuncture) was reported by 61% of patients after 4 months and 66% of patients at 12 months, there were no differences in the rate of concurrent treatment between patients in the work-focused intervention and control intervention (Chi-square 0.36, p = 0.551 at 4 months, and Chi-square 0.03, p = 0.858 at 12 months).

Only 180 (60%) of the patients who completed 12 month follow-up had complete FABQ scores at both baseline and 4 months, and some had only completed one of the subscales (10 missing on FABQ-P subscale and 11 missing on FABQ-W subscale) (Figure 1). Subjects attending a 12-month follow-up with missing FABQ at 4 months did not differ from the subjects with complete FABQ regarding any baseline characteristics except for a higher number of males (66% and 39% respectively, p < 0.001) and blue collar workers (44% and 32% respectively, p = 0.027).

### Change in pain and disability at 12-month follow-up

The mean reduction in pain was 1.59 (SD 2.70) points on NRS in the work-focused intervention and 1.36 (SD 2.88) in the control intervention. For disability, the reduction in ODI/NDI was 8.80 (SD 15.55) in the work-focused intervention and 9.02 (SD 14.67) in the control intervention. The differences in change between the two groups were not statistically significant (Table 3). Analyses with only patients who had complete scores at both measure points (casewise deletion of missing) did not change these results (Table 3).

**Table 3:** **Change in pain and disability from baseline to 12**-**month follow up in work**-**focused intervention and control intervention**

		**Intention to treat analyses (with multiple imputation)**					**Available cases only (Casewise deletion of missing)**				
		**N**	**Mean change**	**SD**	**95% CI for difference**	**p**-**value**	**N**	**Mean change**	**SD**	**95% CI for difference**	**p**-**value**
Pain	Work-focused intervention	201	1.59	2.70	−0.32 to 0.78	0.410	154	1.55	2.73	−1.02 to 0.25	0.230
	Control intervention	197	1.36	2.88			145	1.17	2.82		
Disability	Work-focused intervention	200	8.80	15.55	−3.21 to 2.76	0.881	150	9.24	15.64	−4.71 to 2.28	0.495
	Control intervention	197	9.02	14.67			141	8.02	14.58		

### Association between improvement in fear-avoidance and decreased pain, disability and return to work at 12-month follow-up

FABQ-P and FABQ-W scores decreased in both groups after intervention (4-month follow-up). Improvement in FABQ-P scores after intervention were achieved in 22% (N = 20) of patients in the work-focused intervention and 18% (N = 14) of patients in the control intervention. Improvement in FABQ-W scores were achieved in 26% (N = 24) of patients in the work-focused intervention and 20% (N = 15) in the control intervention. The differences between the groups were not statistically significant (Chi Square; p = 0.362 for FABQ-W, and p = 0.569 for FABQ-P).

Univariate logistic regressions with RTW, pain and disability as response variables were performed and are shown in Table 4. Subsequent multiple logistic regression models including variables with p < 0.2 from the univariate analysis were calculated. The results from the multiple regressions are shown in Tables 5, 6 and 7. All the logistic regression models had acceptable goodness of fit (p-values ranged from 0.118 to 0.952), and none of the predictor variables had a Spearman’s rho above 0.7.

**Table 4:** **Univariate logistic regressions with change in pain at 12**-**months, change in disability at 12**-**months and RTW within 12 months as outcome variables**

	**Pain**				**Disability**				**RTW**			
**Predictors**	**OR**	**95% CI**		**p**-**value**	**OR**	**95% CI**		**p**-**value**	**OR**	**95% CI**		**p**-**value**
Gender	1.29	0.82	2.04	.273	1.17	0.72	1.90	.534	0.99	0.60	1.63	.971
Age	0.97	0.95	0.99	.007**	0.98	0.96	1.00	.115*	0.97	0.94	0.99	,019*
Intervention	1.20	0.76	1.90	.425	1.03	0.63	1.68	.898	0.84	0.51	1.38	.489
Mother tongue	0.71	0.40	1.29	.266	0.55	0.28	1.09	.086*	1.02	0.53	1.94	.958
Lower education	REF				REF				REF			
Medium education	1.44	0.71	2.93	.317	0.97	0.46	2.04	.937	0.75	0.34	1.66	,484
Higher education	1.37	0.63	2.95	.425	0.90	0.40	2.01	.793	0.99	0.42	2.34	,981
low skilled blue collar	REF				REF				REF			
high skilled blue collar	0.95	0.44	2.03	.889	1.13	0.50	2.54	.771	0.80	0.34	1.86	.605
low skilled white collar	0.96	0.48	1.94	.915	0.78	0.37	1.67	.527	1.05	0.48	2.32	.903
high skilled white collar	0.97	0.47	2.00	.932	1.15	0.54	2.48	.714	0.78	0.35	1.74	.547
Improvement in FABQ-W^a^	2.46	1.18	5.10	.016**	2.70	1.23	5.92	.013**	3.33	1.30	8.51	.012**
Improvement in FABQ-P^b^	1.81	0.85	3.86	.125*	3.18	1.40	7.22	.006**	1.77	0.74	4.22	.196*
Derpession score at baseline	1.03	0.96	1.09	.420	0.99	0.93	1.06	.801	0.97	0.91	1.04	.357
Anxiety score at baseline	1.04	0.98	1.10	.220	,983	0.92	1.05	.603	0.93	0.87	0.99	.032**

^a^Fear avoidance beliefs of work, ^b^Fear avoidance beliefs of physical activity, **significant at level p < 0.05, *significant at level p < 0.2.

**Table 5:** **Multivariate logistic regression with improvement in pain (≥2 points NRS) scores at 12**-**month follow**-**up as outcome variable (n = 159)**

**Predictors**	**OR**	**95% C.I.for OR**		**p**-**value**
		**Lower**	**Upper**	
Age	0.98	0.95	1.02	0.318
Men (Women as reference)	1.03	0.53	1.99	0.933
Work-focused intervention	1.04	0.54	2.00	0.917
Improvement in FABQ-W^a^ (no improvement as reference)	2.08	0.92	4.70	0.080
Improvement in FABQ-P^b^ (no improvement as reference)	1.63	0.70	3.79	0.253

Nagelkerke R^2^: 0.07, Cox & Snell R^2^: 0.05.

^a^Fear avoidance beliefs of work, ^b^Fear avoidance beliefs of physical activity.

**Table 6:** **Multivariate logistic regression with improvement in disability (≥12 points ODI/NDI) scores at 12**-**month follow**-**up as outcome variable (n = 156)**

**Predictors**	**OR**	**95% C.I.for OR**		**p**-**value**
		**Lower**	**Upper**	
Age	0.95	0.91	0.99	0.008*
Men (Women as reference)	0.62	0.27	1.42	0.256
Work-focused intervention	0.86	0.38	1.96	0.726
Foreign mother tongue	0.63	0.21	1.90	0.409
Improvement in FABQ-W^a^ (no improvement as reference)	1.72	0.70	4.21	0.234
Improvement in FABQ-P^b^ (no improvement as reference)	3.65	1.43	9.28	0.007*

Nagelkerke R^2^: 0.18 and Cox & Snell R^2:^ 0.12.

^a^Fear avoidance beliefs of work, ^b^Fear avoidance beliefs of physical activity, *significant at level p < 0.05.

**Table 7:** **Multivariate logistic regression with return to work within 12 months as outcome variable (n = 159)**

**Predictors**	**OR**	**95% C.I.for OR**		**p**-**value**
		**Lower**	**Upper**	
Age	0.96	0.92	1.00	0.031*
Men (Women as reference)	0.80	0.39	1.66	0.552
Work-focused intervention	0.97	0.47	1.99	0.926
Improvement in FABQ-W^a^ (no improvement as reference)	3.60	1.19	10.88	0.023*
Improvement in FABQ-P^b^ (no improvement as reference)	1.37	0.50	3.77	0.537
Anxiety score at basline (HADS-A^c^scale)	0.89	0.80	0.98	0.018*
Nagelkerke R^2:^ 0.17, Cox & Snell R^2:^ 0.12.				

^a^Fear avoidance beliefs of work, ^b^Fear avoidance beliefs of physical activity, ^c^Hospital anxiety and depression scale, anxiety subscale *significant at level p < 0.05.

Age and improvement in FABQ-P and FABQ-W scores at 4 months were identified as possible predictors for reduction in pain scores at 12 month follow-up in the univariate analysis. None of these remained significant in the multiple regressions models (Table 5).

Age, mother tongue, and improvement in FABQ-P and FABQ-W scores at 4 months, were identified as possible predictors for reduced disability (p <0.2). In the multiple regression analyses, younger age and improvement in FABQ-P remained positive predictors for improvement in disability (Table 6). Controlling for FABQ-P score at baseline lowered the OR for FABQ-P to 2.7, p = 0.056.

From the univariate analyses with RTW as response variable, age, anxiety score (HADS-A), improvement in FABQ-P and FABQ-W scores at 4 months were possible predictors (p < 0.2). Younger age, low anxiety score and improvement in FABQ-W remained positive predictors in the multiple regression analyses (Table 7). Controlling for baseline values of FABQ-W did not change this result, and the OR for RTW increased to 4.0 (p = 0.015) for the group with improvement in FABQ-W scores.

## Discussion

Pain and disability decreased in both the work-focused and multidisciplinary treatment groups, and no differences were found between them. The short work-focused intervention had similar effect as previously documented multidisciplinary rehabilitation and brief education interventions [7]. The effect on pain was similar to that reported in other studies on work rehabilitation, brief intervention, or multidisciplinary treatment [7,45-47]. The 9-point reduction in the disability score (ODI/NDI) was also similar to other studies on multidisciplinary rehabilitation [46,48,49]. The minimal clinically relevant change in ODI was estimated to be approximately 13 in a recent study [50], and the majority of patients in our study had changes below this threshold. A review of physical and rehabilitation interventions found a moderate level of evidence for a short-term effect on pain and disability with multidisciplinary treatment. However, that review concluded that the differences were small and not clinically relevant [51], and this is in accordance with our results. Whether subgroups of patients benefit more from this type of treatment strategy is still unclear and requires further study. We included both neck and back pain patients in this study, but the number of neck pain patients was too low to do subgroup analyses based on this variable. Therefore the results in this study are primarily valid for back pain patients. Studies have shown, though, that multisite pain is the most common in chronic pain patients [52] and more than half of back pain patients also have concurrent neck pain [52,53]. The management of neck and low back pain also share many commonalities [54], and we believe the results may have relevance for neck pain patients as well.

The length of treatment utilised for chronic low back and neck pain varies. However, a recent Cochrane review concluded with no difference in effect between more and less intensive interventions [55]. This conclusion also seems valid for patients in specialist care [14]. The results from our study indicate that adding work-focus in specialist care does not result in better effect of interventions, but also that a work-focused intervention is not inferior to interventions that focus on physical activity and pain. More research regarding the needed length and intensity of the components in the interventions is needed.

As the work-focused intervention had a specific aim of reducing FAB of work whereas the multidisciplinary interventions were more focused on reduction of FAB of physical activity, we expected a larger reduction in FABQ-W scores in the work-focused intervention. On the contrary, there was a similar reduction of scores in both study groups at the 4 month follow-up, and approximately 20% of patients in both groups had improved scores. This is similar to the effect found in another study in an occupational therapy setting [31]. Other studies of multidisciplinary treatments without work focus have also found an effect on reduced FABQ-W scores [56]. A possible explanation may be that patients transfer an experience of increased coping in physical activities to work-related activities. In addition, other mandatory actions, such as meetings with the employer and social security (NAV) representatives, may have the same effect on facilitating contact with the employer as the case manager in our study.

FAB has been established as an important predictor for outcome in patients with low back pain, and also a possible mediator [24,33]. We found that improved FABQ-W scores after treatment predicted RTW within 12-month follow-up. Patients with 12-points or more improvement in FABQ-W scores had an OR of approximately 3–4 for RTW compared to the group with no change. Similarly, a reduction in FABQ-P was also a positive predictor of reduced disability at 12 months. Our results also indicated that the change in FABQ-W during the interventions is most important for successful RTW, whereas high baseline FABQ-P must be considered as a negative predictor on improvement in disability. Improvement in either of these two scores was not significantly associated with reduction in pain at follow up.

High FABs are documented predictors of both chronicity of pain and failure to RTW [25,57]. Conflicting evidence exists regarding changes in FABs as mediators of treatment outcome [31,56,58]. Our findings support the theory that one mechanism in rehabilitation is the reduction in FABs, and particularly reduction in FABs regarding work is an important predictor for positive outcome.

Unfortunately, our work-focused intervention was not superior to the control intervention in reducing FABs about work, and better treatment modalities are still needed. However, the findings support the importance of addressing work-related issues in multidisciplinary interventions.

### Strengths and limitations

The study was part of a randomised controlled study with relatively large number of included patients, and was conducted in specialist care. The interventions were carried out at two different hospitals localized in different parts of Norway, and this increased external validity of the results. The results from the analyses on pain and disability is reports from the randomised controlled trial, but the two intervention groups were merged in the analyses of FABQ because of no difference in effect between the two treatments groups. The sample size calculation for this study was based on survival analysis of RTW, and not on change in disability and pain. Post hoc analyses of power showed a power above 90% for detecting a difference in pain scores of 1.0 with SD of 2.5 and a difference in disability of 10 with SD of 17 even with 30% drop-out. The back pain cohort in this study was also large compared to many other studies on pain and disability [34,46,47].

The loss to follow-up was large, particularly for the subgroup analysis of FABQ scores. Analyses of the patients lost to follow-up at 4 months showed no significant differences from patients lost to follow-up at 12 months on any of the baseline variables.

The FABQ was used to measure the effect of intervention, but no agreement exists regarding the cut-off for clinically relevant change [59]. We used the minimal detectable change reported in previous studies [44] as the cut-off, and it seemed to predict important differences between patients in our study.

Blinding participants and the treatment team was not possible. The researchers were part of the treatment team, but they were blinded for allocation to avoid assessment bias. An independent researcher revealed the allocation code after the analyses were run. The first part of the brief intervention was carried out before randomization and could be considered as blinded.

This study was conducted in two hospitals in different regions of Norway. The usual treatment used as control interventions in this study differed between the two study sites, but studies have shown similar effect of these two treatment modalities used in specialist care previously [14,60].

To investigate the effect of study site on outcome we carried out post hoc linear regression analyses with study site, intervention and the interaction between these two variables as predictor variables, and change in pain and change in disability as outcome variables. We found no significant effect of study site on change in pain (B = − 0.05, p = 0.910) or disability (B = −3.48, p = 0.106), and no effect of the interaction between study site and interventions for change in pain (B = 1.01, p = 0.070) or disability (B = −5.79, p = 0.057). The R square for the regressions was below 0.02.

The external validity may be deemed better in a multicentre study. A main challenge, though, is the parallel work-focused and control interventions within the same hospital. Contamination of the control intervention with a RTW focus cannot be ruled out, although we tried to use different teams for the interventions.

## Conclusion

The work-focused intervention had the same effect on pain and disability as control interventions. No differences were found in changes in FABs about work between the work-focused and control interventions, but improvement in FABs about work seem to be an important predictor for positive outcome in both groups.

## Abbreviations

RTW: Return to work
FABQ: Fear-Avoidance Belief Questionnaire
FABQ-W: FABQ work subscale
FABQ-P: FABQ physical activity subscale
FABs: Fear-avoidance beliefs
ISCO-88: International standard classification of occupations
NRS: Numeric rating scale
NDI: Neck disability index
ODI: Oswestry disability index
HADS: Hospital anxiety and depression scale
HADS-A: HADS anxiety subscale
HADS-D: HADS depression subscale
NAV: Norwegian labour and welfare administration
AAP: Work assessment allowance
OR: Odds ratio
SD: Standard deviation
